# Calculation of Oxygen Uptake during Ambulatory Cardiac Rehabilitation

**DOI:** 10.3390/jcm13082235

**Published:** 2024-04-12

**Authors:** Holger Stephan, Nils Klophaus, Udo F. Wehmeier, Fabian Tomschi, Thomas Hilberg

**Affiliations:** Department of Sports Medicine, University of Wuppertal, Moritzstraße 14, 42117 Wuppertal, Germany

**Keywords:** VO_2_, oxygen uptake, gas exchange, equations, estimate, cardiac rehabilitation, patients, CPET, cardiopulmonary exercise testing, spiroergometry, cycling

## Abstract

**Background**: Cardiopulmonary exercise testing is not used routinely. The goal of this study was to determine whether accurate estimates of VO_2_ values can be made at the beginning and at the end of a rehabilitation program. **Methods**: A total of 91 cardiac rehabilitation patients were included. Each participant had to complete cardiopulmonary exercise testing at the beginning and at the end of a rehabilitation program. Measured VO_2_ values were compared with estimates based on three different equations. **Results**: Analyses of the means of the differences in the peak values showed very good agreement between the results obtained with the FRIEND equation or those obtained with a combination of rules of thumb and the results of the measurements. This agreement was confirmed with the ICCs and with the standard errors of the measurements. The ACSM equation performed worse. The same tendency was seen when considering the VO_2_ values at percentage-derived work rates. **Conclusions**: The FRIEND equation and the more easily applicable combination of rules of thumb are suitable for estimating the peak VO_2_ and the VO_2_ at a percentage-derived work rate in cardiac patients both at the beginning and at the end of a cardiac rehabilitation program.

## 1. Introduction

Cardiopulmonary exercise testing (CPET) is an instrument used to ascertain peak oxygen uptake (VO_2_) and the degree of impairment, estimate risks (e.g., mortality), assess interventions (e.g., training), reveal drivers of restricted exercise capability and dyspnea, check for coexisting cardiovascular diseases (e.g., ischemic heart disease), and help design training programs [1]. It can be used in patients, healthy people, or athletes, and contrary to ordinary exercise testing, an accompanying gas exchange analysis is performed [2]. Peak VO_2_ can be used to identify exercise intolerance [1]. It shows the contribution of aerobic metabolism to energy consumption [3], allows assessment of exercise capacity [4], and is one of several parameters available to derive training intensity [5,6]. The regular VO_2_ increase is 8.5 to 11 mL∙minute^−1^ per watt [7]. If 8 mL∙min^−1^ per watt is not exceeded, abnormal aerobic capacity must be assumed [4]. In patients with cardiovascular disorders, different VO_2_ kinetics have been described. Progressivity can be lower, flattening can occur after normal progression, and VO_2_ can decrease after an initial increase [8]. Despite the applications and benefits described, CPET does not appear to be a standard diagnostic instrument at all cardiac rehabilitation centers [5]. This is driven by the costs, the need for equipment and expertise, and the time required [9]. In addition, wearing a mask to capture gas exchange is not comfortable for everyone. Moreover, the first ventilatory threshold (VT1), a parameter for the assessment of cardiovascular or muscular limitation [4], is related to peak VO_2_ [5] and can therefore be calculated. Several equations are available to estimate VO_2_ during ergometric cycling [4,10,11]. The accuracy of the FRIEND (Fitness Registry and the Importance of Exercise National Database) equation was shown for healthy subjects [11] and for patients with heart failure [12]. Rules of thumb (ROT), which are very easy to apply, can be used to check the validity of measurements [13]. But according to Nichols et al. [14], the adaptation of peak VO_2_ due to cardiac rehabilitation in patients with coronary heart disease cannot be precisely represented by the ACSM (American College of Sports Medicine) equation. Nevertheless, the authors believe that validation of their results with a greater number of participants is indicated. In addition, estimation of submaximal VO_2_ during exercise appears to be important during the cardiac rehabilitation process to ensure adequate aerobic metabolic stimulus and to permit assessment of energy expenditure under aerobic conditions. 

Therefore, the goal of this study was to determine whether accurate estimates of VO_2_ values at different work rates as well as accurate estimates of peak VO_2_ values can be made using established equations during the rehabilitation periods of cardiac patients. We expected that the FRIEND equation could be used to estimate the values determined at the beginning of a rehabilitation process [12], and similar results were expected for the combination of ROT. The ACSM equation was expected to produce inflated VO_2_ values [12]. However, VO_2_ was underestimated in cyclists using the ACSM equation [15]. Therefore, at the higher performance levels at the end of a rehabilitation period, the ACSM equation might be somewhat more accurate. 

Patients and healthcare professionals could benefit from an equation that allows submaximal VO_2_ and peak VO_2_ to be appropriately estimated, as exercise testing without gas exchange analysis can be performed, which is more cost-effective and associated with fewer requirements without compromising important information related to aerobic metabolism and the derivation of training zones. 

## 2. Materials and Methods

### 2.1. Subjects

Cardiac patients from a local ambulatory rehabilitation and prevention center specializing in cardiology, angiology, and sports medicine were recruited for two separate trials on the effects of a cardiac rehabilitation program. Participation was allowed for male patients with a BMI of 20 to 35 kg/m^2^, aged 40 to 65 years, who had an ejection fraction ≥ 40% and was refused in cases of existing heart failure, implanted pacemakers or defibrillators, complex cardiac arrhythmias, or limitations compromising the ability to use a bicycle. The two trials were authorized by German Pension Insurance (8022-6-NW-Wuppertal-Cardiowell-H-2015 and 8022-6-NW-004-Wuppertal-Berg.Uni 2017-HIIT II-H-2017), approved by the ethics committee of the University of Wuppertal (MS/BB), and realized following the Declaration of Helsinki [16]. Participation was possible only after signing a written informed consent form, which included permission to analyze the data for publication. 

### 2.2. Experimental Design

In both trials, each participant was required to complete CPET at the beginning of the rehabilitation program (pre) and at the end of the rehabilitation program (post). The pre-values of the first trial were merged with the pre-values of the second trial to create one data set. The same procedure was used for the post-values. However, the test data were only considered when exhaustion criteria were met [1,4]. VO_2_ values at percentage-derived work rates obtained using an approach used at the local ambulatory rehabilitation and prevention center to derive exercise intensities (55% of maximal work rate); VO_2_ values attributable to 100 watts, i.e., the intensity at which a blood pressure measurement can be performed to estimate the risk of cardiovascular death [17]; and peak VO_2_ values were used to test the validity of three common and established equations: the ACSM equation [10], the combination of the ROT [13], and the FRIEND equation [11].

### 2.3. Cardiac Rehabilitation and Cardiopulmonary Exercise Testing

All cardiac patients completed a 3-week ambulatory rehabilitation program. Ergometric cycling, which was performed 4–5 days per week for 40 min each, was the most important component. In addition, the rehabilitation program included mobility training, calisthenics training, and endurance-oriented walking. The training was carried out by a specialized trainer and supervised by a medical doctor. 

When the periods began and in the third weeks, at the ends of the rehabilitation periods, CPET was performed on a bicycle ergometer (EC3000; Customed, Ottobrunn, Germany) using breath-by-breath measurement (META-LYZER 3B; CORTEX Biophysik GmbH, Leipzig, Germany), including the recording of a 12-lead ECG (Customed, Ottobrunn, Germany) and automatic blood pressure measurement on the left arm (Customed, Ottobrunn, Germany). As recommended for ambulatory patients and sports rehabilitation, an incremental step test protocol was used, which was characterized by an initial load of 25 watts and an increase in load of 25 watts per 2 min [18]. The patients were instructed to cycle to exhaustion. For premature terminations, at least one criterion [19], e.g., ventricular tachycardia or angina, had to be met. After CPET, patients continued cycling at 25 watts for 2 min. 

### 2.4. Calculations for Estimation of Oxygen Uptake

For each CPET session, the output during the last completed stage, the product of the percentage of the target time achieved during the last stage, and the increment (25 watts) were added to determine the peak work rate achieved during ergometer cycling.

The ACSM equation consists of a rest component, a horizontal component, and a resistance component and is claimed to be most accurate between 50 and 300 watts [10].

3.5 + 3.5 + (1.8 × work rate × 6.12)/body mass

One of the ROT presented by Winkert and Kirsten [13] is used to check the plausibility of the VO_2_ determination at rest, the other is used to check the value during exercise.

Resting condition: 5 mL∙min^−1^∙kg^−1^

Aerobic capacity: 10 mL∙min^−1^∙watt^−1^

Combination: 5 × bodyweight + 10 × work rate

The FRIEND equation was developed to more precisely represent peak VO_2_ during cycling. Gender-specific equations were established, as they perform slightly better [11].

1.74 × (work rate × 6.12/body weight) + 3.5

Men: 1.76 × (work rate × 6.12/body weight) + 3.5

Women: 1.65 × (work rate × 6.12/body weight) + 3.5

### 2.5. Statistical Analysis

Means and standard deviations were used to describe the patient population and summarize the performance data. For the comparisons of the VO_2_ measurements with the equations, Bland–Altman plots were generated; means of the differences (MD), the upper limits of agreement (LoA), and the lower LoA were determined (without using them to generate Bland–Altman plots); intraclass correlation coefficients (ICCs) were determined using the two-way mixed model and based on absolute agreement; and standard errors of measurements ($SEM=standard\;deviation\times \sqrt{1-ICC}$) were calculated. The measured VO_2_ values were used as minuends for the determination of differences between the measured values and estimated values. Due to the large sample size, the distributions were evaluated using Q-Q plots. 

## 3. Results

### 3.1. Subjects

Data from a total of 91 patients with different cardiovascular diseases were considered in this study, and 83 of them achieved at least 100 watts (Table 1). The mean body mass index was elevated (category: overweight) whether the values of the entire collective or only the values of the patients who achieved at least 100 watts were considered [20]. 

### 3.2. Evaluations of Peak Work Rates

For comparisons, data from 182 CPET sessions were considered. Pre-performances ranged from very low to above average (40 to 49 years: <10th to >70th percentile; 50 to 59 years: <10th to >80th percentile; 60 to 69 years: <10th to >80th percentile) [21]. The mean post-values of the performance-indicating parameters were significantly higher compared to the pre-values (Table 2). In both CPET sessions, the patients’ efforts were appropriately high, according to the selected objective criteria [4] and the patients’ perceptions. The change in VO_2_ due to the rehabilitation measures was not different from the changes calculated with the equations. 

**Figure 1:**
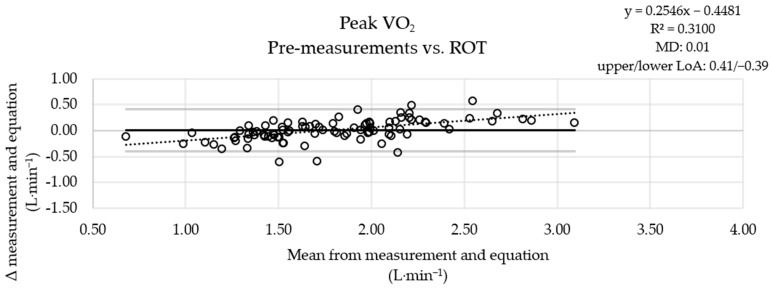
Graphical comparison of the oxygen uptake values of the pre-testing sessions with the ROT combination results (*n* = 91). VO_2_: oxygen uptake; ROT: rules of thumb; upper solid line: upper limit of agreement (LoA); middle solid line: mean of differences (MD) between measurements and equation results; lower solid line: lower limit of agreement; dashed line: linear trend (y: equation of the line; R^2^: determination coefficient).

**Figure 2:**
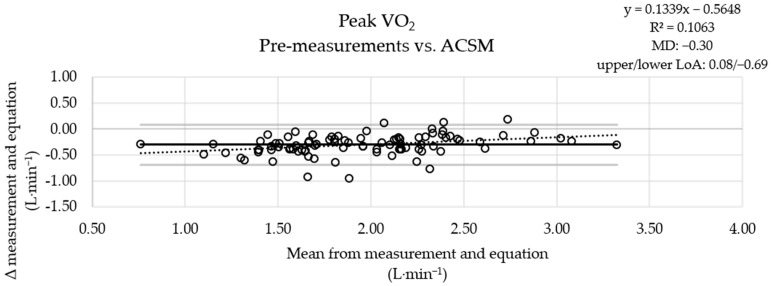
Graphical comparison of the oxygen uptake values of the pre-testing sessions with ACSM equation results (*n* = 91). VO_2_: oxygen uptake; ACSM: American College of Sports Medicine; upper solid line: upper limit of agreement (LoA); middle solid line: mean of differences (MD) between measurements and equation results; lower solid line: lower limit of agreement; dashed line: linear trend (y: equation of the line; R^2^: determination coefficient).

**Figure 3:**
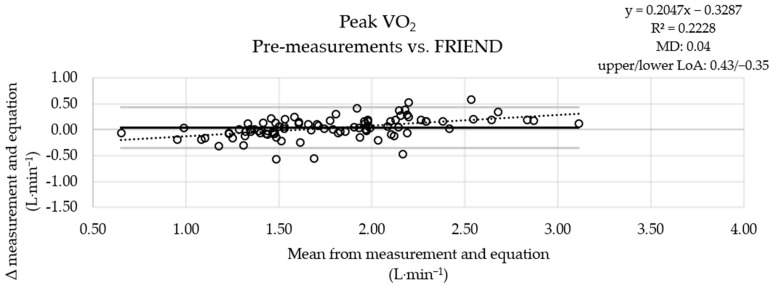
Graphical comparison of the oxygen uptake values of the pre-testing sessions with FRIEND equation results (*n* = 91). VO_2_: oxygen uptake; FRIEND: Fitness Registry and the Importance of Exercise National Database; upper solid line: upper limit of agreement (LoA); middle solid line: mean of differences (MD) between measurements and equation results; lower solid line: lower limit of agreement; dashed line: linear trend (y: equation of the line; R^2^: determination coefficient).

The MD between the measured pre-values and the results of the ROT combination was small (Figure 1), the MD obtained using the ACSM equation was more pronounced (Figure 2), and the results obtained with the FRIEND equation (Figure 3) were similar to those obtained using the ROT combination. An analysis of the measured post-values and the results obtained with the equations showed a similar pattern (Figures S1–S3). 

The intraclass correlation between the peak VO_2_ values attributable to the pre-measurements and the VO_2_ values estimated with the FRIEND equation and that between the peak values and the values estimated with the ROT combination were excellent, while only a good correlation with a wide confidence interval was found with the ACSM equation [22]. The SEM from the comparison of the measured values with the estimates from the ROT combination and that from the comparison with the estimates from the FRIEND equation were similar, while the SEM from the comparison with the estimates from the ACSM equation was slightly higher. The analysis of the values obtained at the ends of the rehabilitation periods showed the same pattern (Table 3).

### 3.3. Evaluations of Fixed Work Rates

From all 182 CPET sessions (pre- and post-tests), the VO_2_ values at percentage-derived work rates were determined. In total, 83 patients achieved at least 100 watts in both CPET sessions (Table 2), and VO_2_ could be determined at this load. An analysis of the differences at fixed work rates (100 watts or 55% of the maximal work rate) showed agreement when the results of the combination of the ROT or those of the FRIEND equation were compared with the pre-values (Table S1). An analysis using the post-values yielded similar results.

The intraclass correlation between the VO_2_ values at percentage-derived work rates attributable to the pre-measurements and the values estimated with the FRIEND equation and that between the values at percentage-derived work rates and the values estimated with the ROT combination were excellent. Only a good correlation with a wide confidence interval was detected using the ACSM equation [22]. The SEM from the comparison of the measured values with the estimates from the combination of the ROT and that from the comparison with the estimates from the FRIEND equation were similar. Applying the ACSM equation resulted in a higher SEM. The analysis of the post-values showed a similar pattern. However, when applying the FRIEND equation, a lower category was achieved with the post-values than with the pre-values (Table 4). The intraclass correlations between the VO_2_ values at 100 watts attributable to the pre-measurements and the estimated VO_2_ values were weak. The SEM for the comparison of the measurements with the estimates from the combination of the ROT and that for the comparison of the measurements with the estimates from the FRIEND equation were similar. Applying the ACSM equation resulted in a higher SEM. The analysis of the post-values showed the same pattern (Table 4).

## 4. Discussion

### 4.1. Highlights

For cardiac patients, the combination of the ROT or the FRIEND equation, respectively, can be applied to estimate peak VO_2_ and VO_2_ at 55% of the maximal work rate. The rehabilitation progress of the patients has no influence on the accuracy of the values determined by these equations.

### 4.2. Classifications

A recent study by Kokkinos et al. [12] with two different cohorts with heart failure demonstrated the predominance of the FRIEND equation compared with the ACSM equation, which distinctly overestimated VO_2_. However, the application of the ACSM equation, which is used for cyclists, by Jurov et al. [15] resulted in a significant underestimation of VO_2_. Therefore, the suitability of these equations presumably depends on the initial performance level. According to Nichols et al. [14], in the context of cardiac rehabilitation, the ACSM equation could not adequately track the change in VO_2_ because it did not correspond to the significant increase in the work rate. Accordingly, the applicability of the equations also likely depends on the training loads or metabolic pathways involved (aerobic vs. anaerobic) as well as the stage of adaptation when performing CPET. Additionally, there are different results related to the influence of the cycling cadence on VO_2_ [23]. Buchanan and Weltman [23] showed that both maximal VO_2_ and VO_2_ at 4 mmol/L decreased with an increasing cadence. In a study by Marsh and Martin [24], higher cadences resulted in higher aerobic demands at various constant loads. It should be considered that in patients with cardiovascular disorders VO_2_ kinetics may be conspicuous [8]. The relation between the work rate and VO_2_ may therefore be compromised [14]. 

### 4.3. Applications

CPET provides more information compared to other exercise tests and can be useful not only for detecting diseases but also for determining pathophysiology [4]. The equations can be considered as a supplement to CPET and not as a surrogate, especially in patients. If VO_2_ is determined with CPET, the estimated value can be used comparatively. Subsequently, a selected equation can be applied for further performance determinations, e.g., after training periods, if no other parameters that are usually determined using CPET are required in addition to VO_2_. In particular, the FRIEND equation and the combination of the ROT are suitable for estimating VO_2_ values in cardiac patients. However, the combination of the ROT is much easier to use because of its simple structure, which allows quick calculations without technical devices. Of course, performance can also be assessed using the peak power output achieved on a cycle ergometer or the maximal velocity achieved on a treadmill. However, extensive and high-quality overviews with standard VO_2_ values are available [21], which allow the classification of individual results. In addition, performances determined in different disciplines can be compared with each other if VO_2_ values have been determined. Based on the MD, very good agreement between VO_2_ values determined via equations and measured VO_2_ values can be seen; however, individual deviations may occur. It should be taken into account that under certain medical circumstances maximal effort cannot be achieved [2]. In addition, there are individual reasons to forgo maximal utilization (e.g., sweating, exhaustion, and lack of time). In these cases, the VO_2_ values at submaximal levels can be calculated and related to the values obtained in previous tests to estimate in which ranges, formed by percentages of peak VO_2_ [5] or percentages of VO_2_max [2], the exercises are taking place. The complementary use of equations can save costs, avoid the potential inconvenience of wearing a mask, and save time. In addition, decentralized analysis of performance and internal load independent of medical facilities with CPET equipment is possible.

### 4.4. Limitations

With this substantial and comprehensive work, we have shown that in ambulatory cardiac patients both peak VO_2_ and VO_2_ at a percentage-derived work rate can be estimated with different equations and that rehabilitation measures do not affect the accuracy of the equations. However, some limitations must be acknowledged. Although there are very few data related to estimating changes in VO_2_ due to ambulatory cardiac rehabilitation measures in women [14], only men were included. Data from 182 CPET sessions with ambulatory cardiac patients could be included. However, with a higher number, the explanatory power would be even greater. In addition, the fitness levels of the patients were different. Therefore, the difficulty of a work rate of 100 watts was variable among the patients. Although the use of equations assumes a linear increase in VO_2_ proportional to the increase in wattage, the kinetics may be different in patients. In addition, it has to be considered that cardiac rehabilitation in Germany usually lasts only three weeks and that a longer training period will most likely lead to larger adaptations. This could affect the validity of the equations. 

## 5. Conclusions

CPET is an instrument used to ascertain peak VO_2_ and the degree of impairment, estimate risks, assess interventions, reveal drivers of restricted exercise capability and dyspnea, check for coexisting cardiovascular diseases, and help design training programs. However, its application requires equipment, is costly and time-consuming, and requires expertise. If VO_2_ is required, various equations are available. With the FRIEND equation and the combination of ROT, peak VO_2_ values and VO_2_ values at percentage-derived work rates can be estimated in cardiac patients at the beginning and at the end of an ambulatory cardiac rehabilitation program. Further research should address the impacts of longer rehabilitation periods. 

## Figures and Tables

**Table 1:** Patient characteristics (*n* = 91) for the categories of core data, diseases, and medication (mean ± standard deviation).

Characteristics	
**Core data**	
Age (years)	53.7 ± 5.9
Height (cm)	178.2 ± 7.1
Body mass (kg)	87.6 ± 12.9
BMI (kg∙m^−2^)	27.6 ± 3.9
**Prevalences of diseases**	
I10 Essential (primary) hypertension	50
I11 Hypertensive heart disease	28
I21 Acute myocardial infarction	56
I24 Other acute ischemic heart diseases	13
I25 Chronic ischemic heart disease	81
I34 Nonrheumatic mitral valve disorders	17
I35 Nonrheumatic aortic valve disorders	6
I36 Nonrheumatic tricuspid (valve) disorders	3
Z95 Presence of cardiac and vascular implants and grafts	83
**Medication**	
Platelet aggregation inhibitors	92
Statins	92
Beta blockers	61
Antihypertensives	28
ACE inhibitors	65
Other medications	62

BMI: body mass index; ACE: angiotensin-converting enzyme.

**Table 2:** Results of the cardiopulmonary exercise testing (mean ± standard deviation).

Performance Capability		Peak WR (watt)	Peak WR (watt∙kg^−1^)	Peak VO_2_ (L∙min^−1^)	Peak VO_2_ (mL∙min^−1^∙kg^−1^)
Entire patient collective (*n* = 91)	pre post	135.8 ± 37.7 152.7 ± 41.7 *	1.56 ± 0.42 1.76 ± 0.47 *	1.81 ± 0.52 1.97 ± 0.56 *	20.7 ± 5.3 22.6 ± 6.1 *
Patients who achieved ≥ 100 watts in both CPET sessions (*n* = 83)	pre post	141.4 ± 34.0 158.6 ± 38.2 *	1.62 ± 0.39 1.82 ± 0.44 *	1.88 ± 0.47 2.06 ± 0.51 *	21.4 ± 4.9 23.5 ± 5.6 *

WR: work rate; VO_2_: oxygen uptake; CPET: cardiopulmonary exercise testing; * significant difference (*p* < 0.001) between pre and post.

**Table 3:** Correlations between measured and estimated peak oxygen uptake values and derived standard errors of measurements (*n* = 91).

Peak VO_2_ Values		Measured vs. ROT	Measured vs. ACSM	Measured vs. FRIEND
pre	ICC	0.948 (0.921–0.966)	0.870 (−0.075–0.963)	0.952 (0.927–0.968)
	SEM (L∙min^−1^)	0.10	0.17	0.10
post	ICC	0.959 (0.938–0.973)	0.884 (−0.093–0.969)	0.965 (0.947–0.977)
	SEM (L∙min^−1^)	0.10	0.18	0.10

VO_2_: oxygen uptake; ROT: rules of thumb; ACSM: American College of Sports Medicine; FRIEND: Fitness Registry and the Importance of Exercise National Database; ICC: intraclass correlation coefficient.

**Table 4:** Correlations between measured and estimated oxygen uptake values at fixed work rates (100 watts, *n* = 83; 55% of peak WR, *n* = 91) and derived standard errors of measurements.

VO_2_ Values			Measured vs. ROT	Measured vs. ACSM	Measured vs. FRIEND
pre	100 watts	ICC	0.329 (−0.029–0.564)	0.138 (−0.127–0.391)	0.256 (−0.144–0.517)
		SEM (L∙min^−1^)	0.10	0.13	0.09
	55% of peak WR	ICC	0.924 (0.885–0.950)	0.756 (−0.182–0.927)	0.915 (0.849–0.949)
		SEM (L∙min^−1^)	0.07	0.14	0.08
post	100 watts	ICC	0.379 (0.037–0.600)	0.172 (−0.144–0.450)	0.278 (−0.084–0.523)
		SEM (L∙min^−1^)	0.09	0.12	0.09
	55% of peak WR	ICC	0.903 (0.853–0.936)	0.788 (−0.123–0.931)	0.898 (0.825–0.937)
		SEM (L∙min^−1^)	0.10	0.15	0.10

VO_2_: oxygen uptake; ROT: rules of thumb; ACSM: American College of Sports Medicine; FRIEND: Fitness Registry and the Importance of Exercise National Database; ICC: intraclass correlation coefficient.

## Data Availability

The data are available on request.

## Supplementary Materials

The following supporting information can be downloaded at https://www.mdpi.com/article/10.3390/jcm13082235/s1, Table S1: Comparison of measured oxygen uptake values with estimated values at fixed work rates (100 watts, *n* = 83; 55% of peak WR, *n* = 91); Figure S1: Graphical comparison of the oxygen uptake values of the post-testing sessions with the ROT combination results; Figure S2: Graphical comparison of the oxygen uptake values of the post-testing sessions with the ACSM equation results; Figure S3: Graphical comparison of the oxygen uptake values of the post-testing sessions with the FRIEND equation results.
