# Supplementary material for: Calculation of Oxygen Uptake during Ambulatory Cardiac Rehabilitation
Source: J Clin Med. 2024 Apr 12;13(8):2235. doi: 10.3390/jcm13082235 (PMC11050814; doi:10.3390/jcm13082235)
Supplement: Supplementary file 1 [file jcm-13-02235-s001.zip › jcm-2906891-supplementary.pdf]

Journal of
Clinical Medicine

MDPI

Table S1. Comparison of measured oxygen uptake values with estimated values at fixed work rates (100 watts, n = 83; 55% peak WR, n = 91).

Pre					Post			
Mean VO_2	MD	Upper LoA	Lower LoA	Comparisons	Mean VO_2	MD	Upper LoA	Lower LoA
	(l·min-1)					(l·min-1)		
	−0.03	0.30	−0.36	VO_2 vs. ROT 100 watt		0.00	0.31	−0.32
1.41	−0.31	0.03	−0.64	VO_2 vs. ACSM 100 watt	1.44	−0.28	0.04	−0.60
	0.03	0.36	−0.31	VO_2 vs. FRIEND 100 watt		0.05	0.37	−0.27
	−0.01	0.26	−0.29	VO_2 vs. ROT 55% peak WR		0.00	0.37	−0.36
1.17	−0.27	0.02	−0.55	VO_2 vs. ACSM 55% peak WR	1.28	−0.26	0.10	−0.62
	0.06	0.34	−0.22	VO_2 vs. FRIEND 55% peak WR		0.07	0.43	−0.29

MD: mean of the differences; LoA: limit of agreement; VO_2: oxygen uptake; VO_2c: oxygen uptake determined with equation; WR: work rate; ROT: rules of thumb; ACSM: American College of Sports Medicine; FRIEND: Fitness Registry and the Importance of Exercise National Database

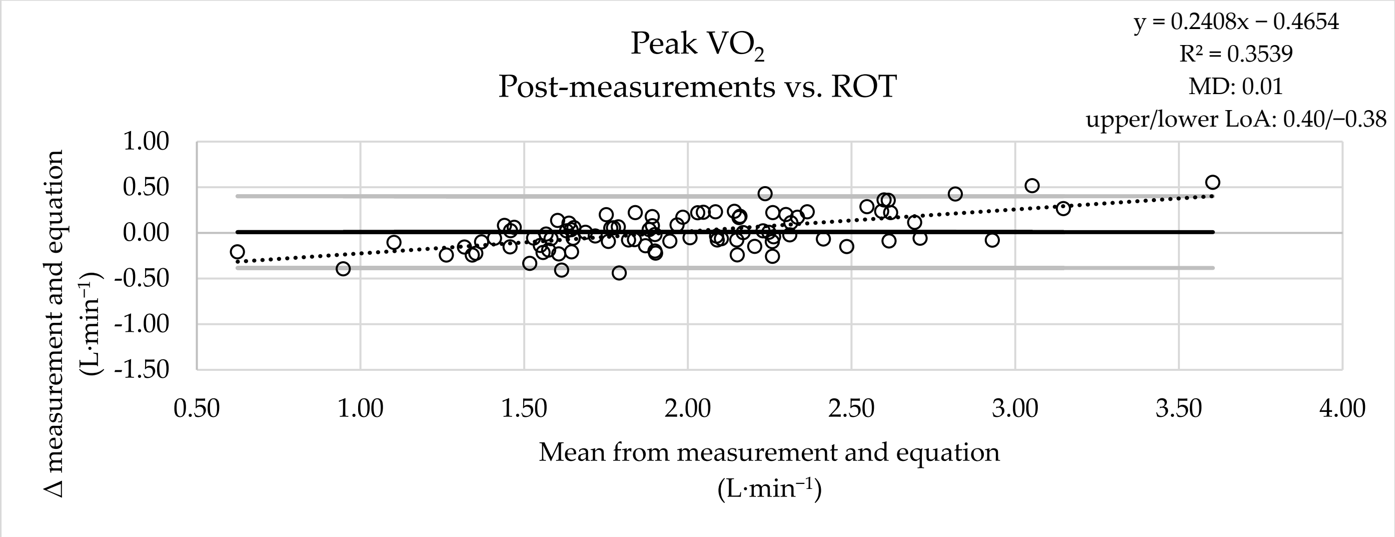

Figure S1. Graphical comparison of the oxygen uptake values of the post-testings with the ROT combination results (n = 91). VO_2: oxygen uptake; ROT: rules of thumb; upper solid line: upper limit of agreement (LoA); middle solid line: mean of differences (MD) between measurements and equation results; lower solid line: lower limit of agreement; dashed line: linear trend (y: equation to the line; R^2: determination coefficient).

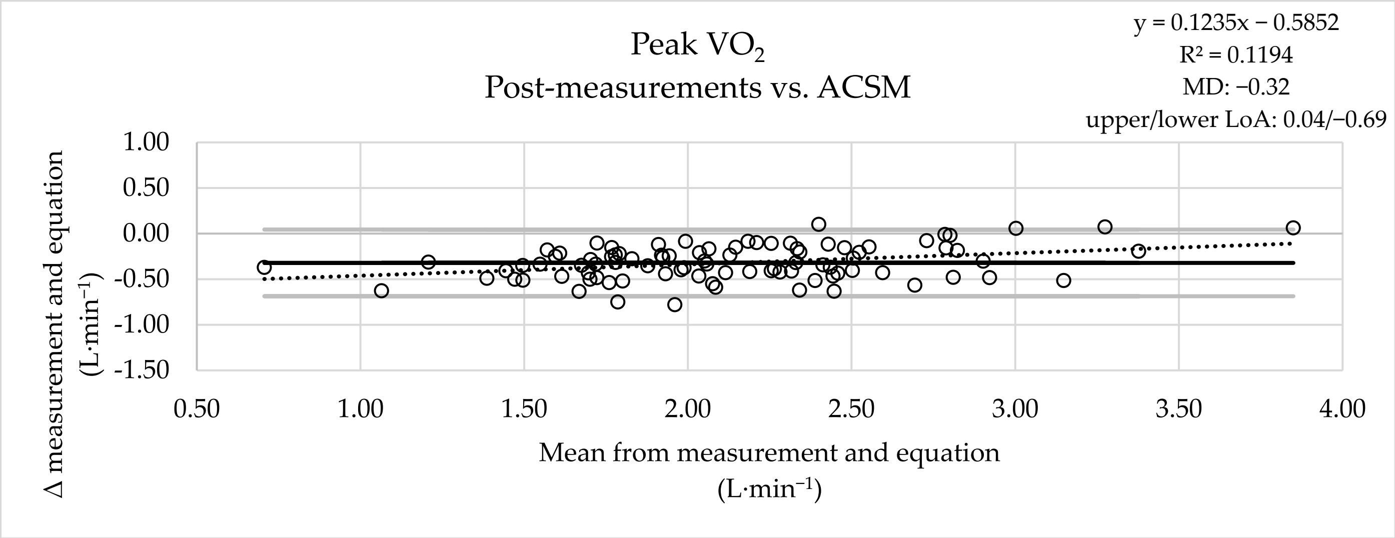

Figure S2. Graphical comparison of the oxygen uptake values of the post-testings with ACSM equation results (n = 91). VO_2: oxygen uptake; ACSM: American College of Sports Medicine; upper solid line: upper limit of agreement (LoA); middle solid line: mean of differences (MD) between measurements and equation results; lower solid line: lower limit of agreement; dashed line: linear trend (y: equation to the line; R^2: determination coefficient).

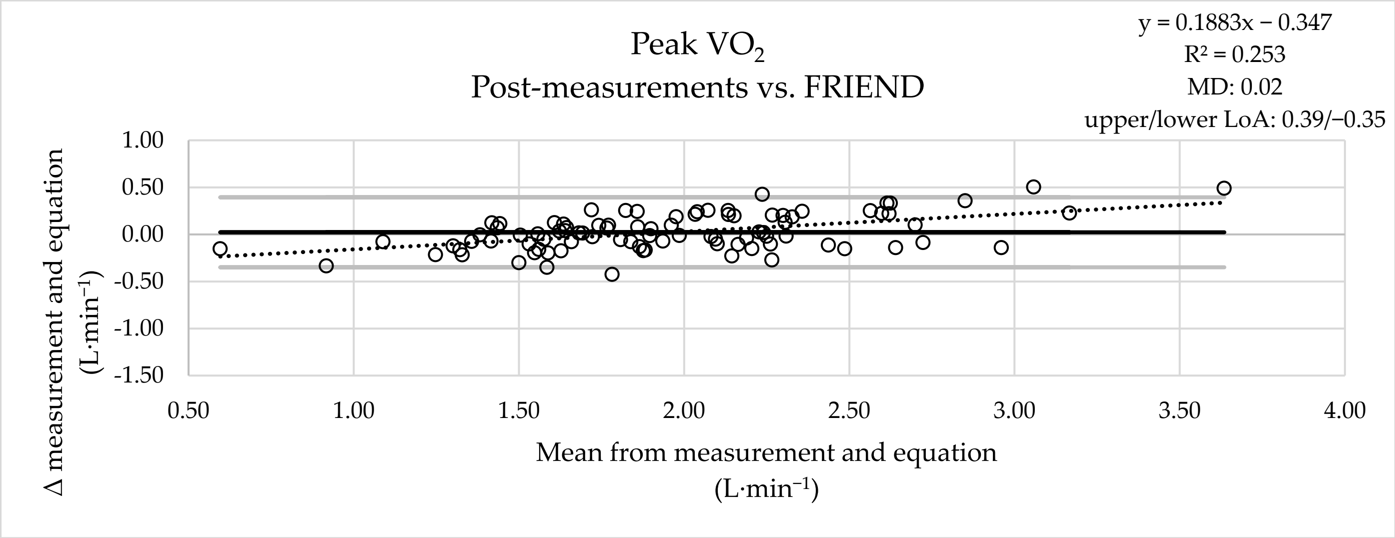

Figure S3. Graphical comparison of the oxygen uptake values of the post-testings with FRIEND equation results (n = 91). VO_2: oxygen uptake; FRIEND: Fitness Registry and the Importance of Exercise National Database; upper solid line: upper limit of agreement (LoA); middle solid line: mean of differences (MD) between measurements and equation results; lower solid line: lower limit of agreement; dashed line: linear trend (y: equation to the line; R^2: determination coefficient).
